# Association of IFN-γ +874 A/T SNP and hypermethylation of the -53 CpG site with tuberculosis susceptibility

**DOI:** 10.3389/fcimb.2023.1080100

**Published:** 2023-01-19

**Authors:** Guadalupe Inés Álvarez, Rodrigo Emanuel Hernández Del Pino, Angela María Barbero, Martín Andrés Estermann, Josefina Celano, Rosa María Musella, Domingo Juan Palmero, Verónica Edith García, Virginia Pasquinelli

**Affiliations:** ^1^ Centro de Investigaciones Básicas y Aplicadas (CIBA), Universidad Nacional del Noroeste de la Provincia de Buenos Aires (UNNOBA), Buenos Aires, Argentina; ^2^ Centro de Investigaciones y Transferencias del Noroeste de la Provincia de Buenos Aires (CIT NOBA), UNNOBA- Universidad Nacional de San Antonio de Areco (UNSAdA) - Consejo Nacional de Investigaciones Científicas y Técnicas (CONICET), Buenos Aires, Argentina; ^3^ Instituto de Inmunología, Genética y Metabolismo (INIGEM), Universidad de Buenos Aires (UBA) – CONICET, Buenos Aires, Argentina; ^4^ División Tisioneumonología Hospital F.J. Muñiz, Buenos Aires, Argentina; ^5^ CONICET-Universidad de Buenos Aires, Instituto de Química Biológica de la Facultad de Ciencias Exactas y Naturales (IQUIBICEN), Buenos Aires, Argentina; ^6^ Universidad de Buenos Aires, Facultad de Ciencias Exactas y Naturales, Departamento de Química Biológica, Buenos Aires, Argentina

**Keywords:** tuberculosis, IFNg, SNPs, methylation, susceptibility

## Abstract

**Introduction:**

Tuberculosis (TB) is now the 2nd leading infectious killer after COVID-19 and the 13th leading cause of death worldwide. Moreover, TB is a lethal combination for HIV-patients. Th1 responses and particularly IFN-γ are crucial for immune protection against Mycobacterium tuberculosis infection. Many gene variants for IFNG that confer susceptibility to TB have been described in multiple ethnic populations. Likewise, some epigenetic modifications have been evaluated, being CpG methylation the major epigenetic mark that makes chromatin inaccessible to transcription factors, thus avoiding the initiation of IFNG transcription.

**Methods:**

We evaluated both genetic and epigenetic changes involved in IFN-γ production and TB susceptibility in Argentine population. Amplification refractory mutation system-polymerase chain reaction (ARMS-PCR) was performed for the IFN-γ +874 A/T polymorphism (rs2430561) genotyping in 199 healthy donors (HD) and 173 tuberculosis (TB) patients. IFN-γ levels from M. tuberculosis-stimulated PBMCs were measured by ELISA. The methylation status at the -53 CpG site of the IFNG promoter in individuals with latent infection (LTBI), TB and HD was determine by pyrosequencing.

**Results:**

Using a case-control study, we found that A allele and, consequently, AA genotype were overrepresented in patients with active disease. Moreover, HD carrying T allele (AT or TT genotype) evidenced an augmented IFN-γ secretion compared to TB patients. Codominance was the genetic model that best fits our results according to the Akaike information criterion (AIC). In addition, increased methylation levels at the -53 CpG site in the IFN-γ promoter were observed in whole blood of patients with active TB compared to LTBI individuals.

**Discussion:**

IFN-γ is regulated by genetic variants and epigenetic modifications during TB. Besides, AA genotype of the rs2430561 single nucleotide polymorphism could be considered as a potential TB susceptibility genetic biomarker in Argentina and the methylation of the -53 CpG site could result in a useful predictor of TB reactivation.

## Introduction

Tuberculosis (TB), a chronic infectious disease caused by the pathogen *Mycobacterium tuberculosis*, remains a major cause of morbidity and mortality worldwide. Until COVID-19 pandemic, TB was the most common cause of death from a single infectious agent, surpassing HIV/AIDS (World Health Organization (WHO), 2022). The impacts generated in 2020 by the pandemic included reduced access to diagnosis and treatment with the consequent increase in the number of deaths. The WHO estimated 1.4 million TB deaths among HIV-negative patients and an additional of 187.000 among HIV-positive people (World Health Organization (WHO), 2022). In Argentina, the last report indicated 10.896 new cases and 656 deaths in 2020 with a rate of mortality of 1.4/100.000 inhabitants (Ministerio De Salud Argentina, 2022). It is estimated that about a quarter of the world’s population has latent TB infection, and is therefore at risk of developing active disease during their lifetime (World Health Organization (WHO), 2022). However, only 5-10% of individuals infected with *M. tuberculosis* will progress to active TB, suggesting that both host genetic and environmental factors might influence the susceptibility to TB (Fortin et al., 2007). Moreover, it has been shown that *M. tuberculosis* sublineages evolved in different human populations showing significant differences in virulence and immunomodulatory functions (Saelens et al., 2019), demonstrating that the genetic variability of *M. tuberculosis* is also an important factor affecting the pathogenesis of the disease. Although most environmental mycobacteria are non-pathogenic species, *M. tuberculosis* has evolved from an opportunist to a professional pathogen capable of infecting and surviving within the host.

Cell-mediated immunity plays an essential role in eliciting a protective immune response against *M. tuberculosis* infection. The secretion of Th1 cytokines by antigen-specific T cells collaborates with protective granuloma formation and stimulates the antimicrobial activity of infected macrophages (Flynn and Chan, 2001). In particular, IFN-γ is a key Th1 cytokine produced primarily by natural killer cells and T cells. IFNG KO mice infected with *M. tuberculosis* fail to produce reactive nitrogen intermediates that restrict the growth of the bacilli (Cooper et al., 1993; Flynn et al., 1993). Genetic studies in families with Mendelian Susceptibility to Mycobacterial Disease (MSMD), a primary immune deficiency that results in partial or complete defects in IFN-γ secretion, production, binding, or signaling, highlighted the critical role of this cytokine in mycobacterial infections (Rosain et al., 2019).

Family-based genetic studies and population-based case-control association analyses have been used to identify candidate genes for susceptibility to tuberculosis. Besides, cytokine gene polymorphisms underlie the complexity of inter-individual differences in the susceptibility, severity and clinical outcomes of several infectious diseases (Cooke and Hill, 2001; Patarčić et al., 2015). In particular, the +874 A/T (rs2430561) single nucleotide polymorphism (SNP) is located at the 5’ end of the CA repeat region in the first intron of the IFN-γ gene (Pravica et al., 2000). Pravica et al. suggested that the functional role of the +874 A/T SNP is related to its location within a putative NF-κB binding site (Pravica et al., 2000). The T allele predisposes to the binding of the transcription factor NF-κB, whereas the A allele reduces the affinity and therefore the expression of the gene in response to stimulus (Pravica et al., 2000). Thus, AA genotype or A allele have been associated with low levels of IFN-γ production, otherwise TT genotype or T allele are associated with higher production of this cytokine, and AT genotype with intermediate levels (Pravica et al., 2000). A significant association between this SNP and TB has been described in several populations around the world, as in the Sicilian, Brazilian, Chinese, Egyptian children, Spanish, Warao indigenous, South Indian and Iranian population (Lio et al., 2002; Lopez-Maderuelo et al., 2003; Tso et al., 2005; Amim et al., 2008; Mosaad et al., 2010; Bhanothu et al., 2015; Beiranvand et al., 2016; Araujo et al., 2017). Moreover, +874 A/T IFNG polymorphism has been associated with TB and extrapulmonary TB susceptibility in meta-analysis studies (Pacheco et al., 2008; Wei et al., 2017; Mandal et al., 2019; Areeshi et al., 2021). However, this SNP has not been studied in TB patients from Argentina.

On the other hand, IFN-γ gene is subject to both genetic and epigenetic modifications. This could imply a critical link between epigenetics and transcription factors in the regulation of IFN-γ and, consequently, in T cells responses elicited against *M. tuberculosis*. Host, pathogen & environment are the triad, which influences any disease, and epigenetics can bridge the gaps between them. The association between different epigenetic modifications and disease progression in *M. tuberculosis* is becoming an area of growing interest (Fatima et al., 2021; Gauba et al., 2021). Epigenetic changes, for example, histone modifications, DNA methylation, and miRNA-mediated up/downregulation of immune genes, play an important role in immunomodulation of the host at the post-TB infective phase. The reversibility of these epigenetic modifications makes them ideal and innovative targets that can be exploited for drug development and control strategies.

In particular, methylation in the promoter region can lead to the failure of gene transcription initiation and gene silencing by hampering transcription factor binding to specific motifs. The proximal region of the IFNG promoter contains binding sites for several transcription factors, including NFAT, NF-κB, and CREB-ATF1. Interestingly, methylation at the -53 site of the IFNG promoter results in inhibition of CREB and ATF2/c-Jun binding to the proximal AP1 site and is sufficient to inhibit expression of this cytokine in a Th1 murine cell line (Jones and Chen, 2006). Moreover, hypermethylation in human Th2 cells results in chromatin condensation and exclusion of CREB proteins from the IFNG promoter (Yano et al., 2003). CREB upregulates IFN-γ production in human T cells that respond to *M. tuberculosis* (Samten et al., 2002; Samten et al., 2005). We have previously shown that costimulation through the Signal Lymphocyte Activation Molecule (SLAM) in *M. tuberculosis*-stimulated T cells induces CREB activation, leading to IFN-γ secretion (Pasquinelli et al., 2009). Thus, our hypothesis was that the methylation of the CpG -53 site of IFNG could lead to the negative regulation of this cytokine during the immune response against *M. tuberculosis*.

The aim of this study was to further characterize the genetic and epigenetic mechanisms that regulate IFN-γ responses during *M. tuberculosis* infection. To this end, we performed a study in patients with active TB and healthy donors (HD) to determine the influence of +874 A/T polymorphism on tuberculosis susceptibility. We additionally evaluated DNA methylation at the -53 CpG site of the promoter region of IFNG as a new mechanism of epigenetic regulation of IFN-γ in the immune response against *M. tuberculosis*.

## Results

### Demographic characteristics of the studied population

In a case-control study we genotyped the IFNG +874 A/T SNP in 199 healthy donors (HD) and 173 tuberculosis (TB) patients enrolled between 2012 and 2016. Demographic characteristics of both populations are shown in **Table 1**. We did not find differences regarding age distribution between HD and TB patients (*P*>0.05). However, we observed differences in sex distribution of recruited individuals of both populations (HD vs TB patients, *P*<0.0001). Nevertheless, we did not find differences in the genotype distributions and allele frequencies between females and males within each population (**Supplementary Table S1**).

**Table 1:** Demographic Characteristics of patients with Tuberculosis (TB) and Healthy Donors (HD).

Characteristic	Groups		*P Value*
	HD	TB	HD vs TB
**Age (Mean; ± SEM)**	33.62 ± 1.08	32.70 ± 1.18	0.5682^a^
**Sex (N; %)**	Total 199	Total 173	
** Male**	68 (34.17)	133 (76.88)	*< 0.0001* ^b^
** Female**	126 (63.32)	26 (15.03)	
** No data**	5 (2.51)	14 (8.09)	
**Ethnicity (N; %)**	Total 199	Total 173	
** Caucasian**	160 (80.40)	79 (45.66)	*< 0.0001* ^b^
** American Indian**	18 (9.05)	61 (35.26)	
** No data**	21 (10.55)	33 (19.08)	

Categorical variables are expressed in percentages. Age value is expressed as mean ± standard error of the mean (SEM). ^a^ *P* values were calculated by Unpaired t test for unpaired samples, ^b^ *P* values were calculated by the Fisher’s exact test for categorical variables.

### Distribution of genotype and allele frequencies of IFN-γ gene (+874 A/T)

In order to investigate the association between +874 A/T SNP of IFNG and tuberculosis population in Argentina, genomic DNA extracted from whole blood and oral swabs in nucleic acid paper was genotyped by ARMS-PCR as described in Material and Methods. All populations were in Hardy-Weinberg (HW) equilibrium, with no significant Chi-square values for observed and expected genotype frequencies for this SNP.

**Figure 1:**
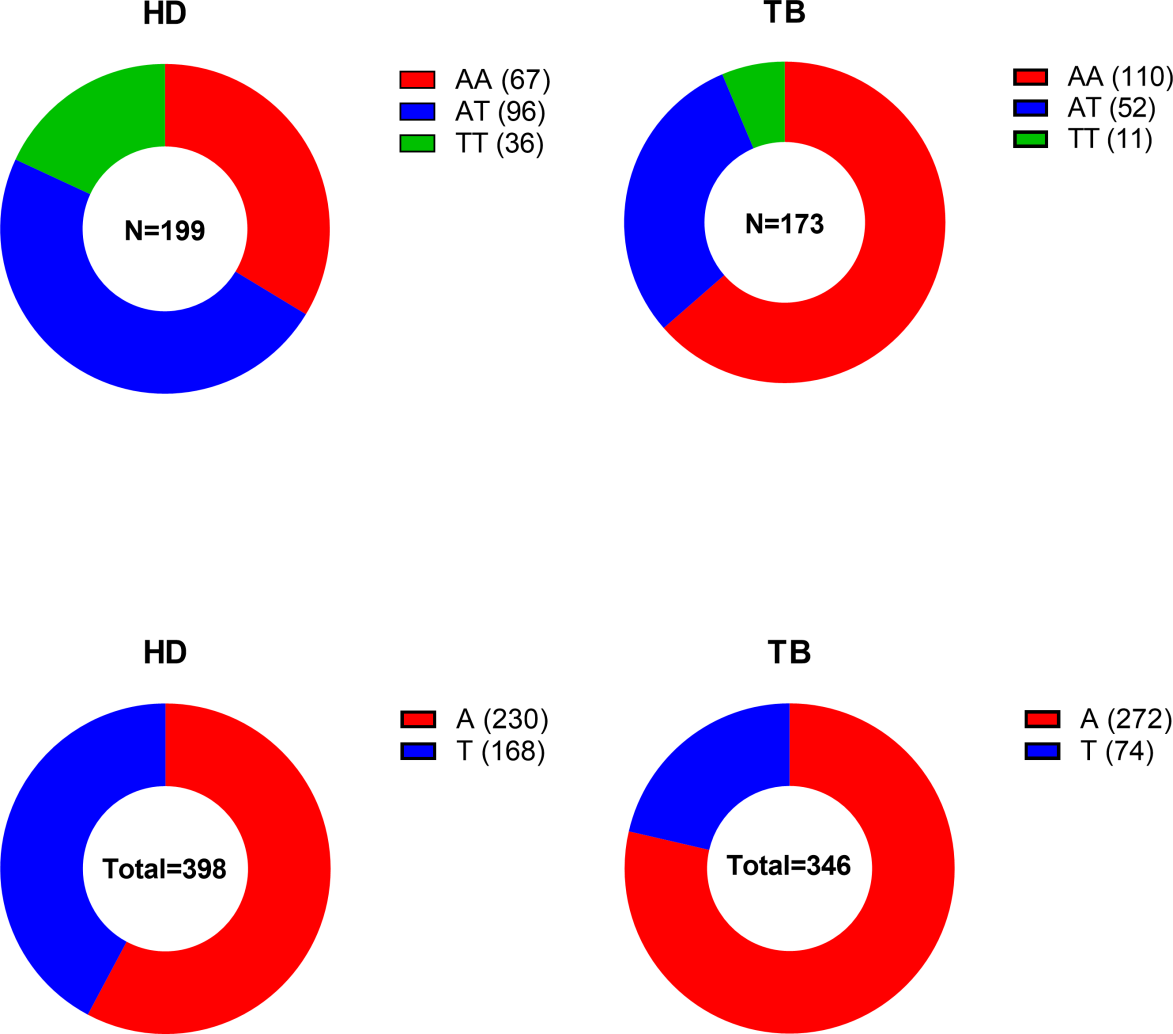
Distribution of genotype and allele frequencies of IFN-γ gene (+874 A/T) among patients with tuberculosis (TB) and Healthy Donors (HD).

The allelic distribution and the genotype of +874 A/T polymorphism differed significantly between TB patients and HD (**Table 2** and **Figure 1**). In fact, the A allele was overrepresented in TB patients (TB= 78.61% and HD= 57.79%; ×^2^ = 36.57 p<0.0001) (**Figure 1** and **Table 2**). Accordingly, the distribution of genotype frequencies between groups was widely significant (TB vs HD ×^2^ = 35.18; p<0.0001). The AA genotype, associated with susceptibility in other populations (Lopez-Maderuelo et al., 2003; Amim et al., 2008; Pacheco et al., 2008; Mosaad et al., 2010; Hashemi et al., 2011), was the most frequent in TB patients (TB= 63.58% and HD= 33.67%), while heterozygous AT genotype was the most frequent in HD (HD= 48.24% and TB= 30.06%) (**Figure 1** and **Table 2**).

**Table 2:** Distribution of genotype and allele frequencies of IFN-γ gene (+874 A/T) among patients with tuberculosis (TB) and Healthy Donors (HD). Categorical variables are expressed in percentages.

		Groups		Chi-square Test *P* Value	HD vs TB OR (95% IC)/*P* Value
		HD	TB	HD vs TB	
**Genotypes**	AA	33.67% (67/199)	63.58% (110/173)	*< 0.0001^a^*	
	AT	48.24% (96/199)	30.06% (52/173)		3.031 (1.925 to 4.773)/< 0,0001^b^
	TT	18.09% (36/199)	6.36% (11/173)		5.373 (2.563 to 11.27)/< 0.0001^b^
**Alleles**	A	57.79% (230/398)	78.61% (272/346)	*< 0.0001^a^*	
	T	42.21% (168/398)	21.39% (74/346)		

^a^ *P* values were calculated by the Chi-Square (χ2) test of homogeneity. ^b^ *P* values were calculated by the Fisher’s exact test for categorical variables.

Odds ratios were calculated to estimate the level of association between the +874 A/T SNP and TB disease. The odds ratio (OR) AA vs AT was 3.031 (95% CI 1.925 - 4.773) and AA vs TT was 5.373 (95% CI 2.563 - 11.27) (**Table 2**). Taken together, these results demonstrate that individuals carrying the AA genotype are more susceptible to develop TB.

An analysis of the codominant, dominant, recessive and over dominant genetic models of inheritance was performed. We found significant differences between healthy controls and patients for the four models studied (**Table 3**). According to the Akaike information criterion (AIC), the codominant model (AA vs AT&TT) was the genetic model that best-fit to our data, since it showed the minimum AIC value (483.9).

**Table 3:** Association studies of IFN-γ gene (+874 A/T) with the development of active tuberculosis.

Model	Genotype	HD	TB	OR (95% CI)	*P value*	AIC	BIC
**Codominant**	A/A	67 (33.7%)	110 (63.6%)	1.00	< 0.0001	483.9	495.6
	A/T	96 (48.2%)	52 (30.1%)	3.03 (1.92 – 4.77)			
	T/T	36 (18.1%)	11 (6.4%)	5.37 (2.56 – 11.27)			
**Dominant**	A/A	67 (33.7%)	110 (63.6%)	1.00	< 0.0001	484.2	492
	A/T - T/T	132 (66.3%)	63 (36.4%)	3.44 (2.24 – 5.27)			
**Recessive**	A/A - A/T	163 (81.9%)	162 (93.6%)	1.00	0.0005	505.7	513.5
	T/T	36 (18.1%)	11 (6.4%)	3.25 (1.60 - 6.61)			
**Overdominant**	A/A - T/T	103 (51.8%)	121 (69.9%)	1.00	0.0003	505	512.8
	A/T	96 (48.2%)	52 (30.1%)	2.17 (1.41 – 3.33)			

### Association of +874 A/T SNP with IFN-γ production and clinical parameters

The A allele and the AA genotype had been associated with low IFN-γ expression and susceptibility to TB in several populations around the world (Lio et al., 2002; Lopez-Maderuelo et al., 2003; Amim et al., 2008; Pacheco and Moraes, 2009; Mosaad et al., 2010; Prabhu Anand et al., 2010; Hashemi et al., 2011). Therefore, we evaluated the association between the IFNG +874 A/T genotypes and the levels of this cytokine produced by *M. tuberculosis*-stimulated PBMCs from HD and TB patients.

**Figure 2:**
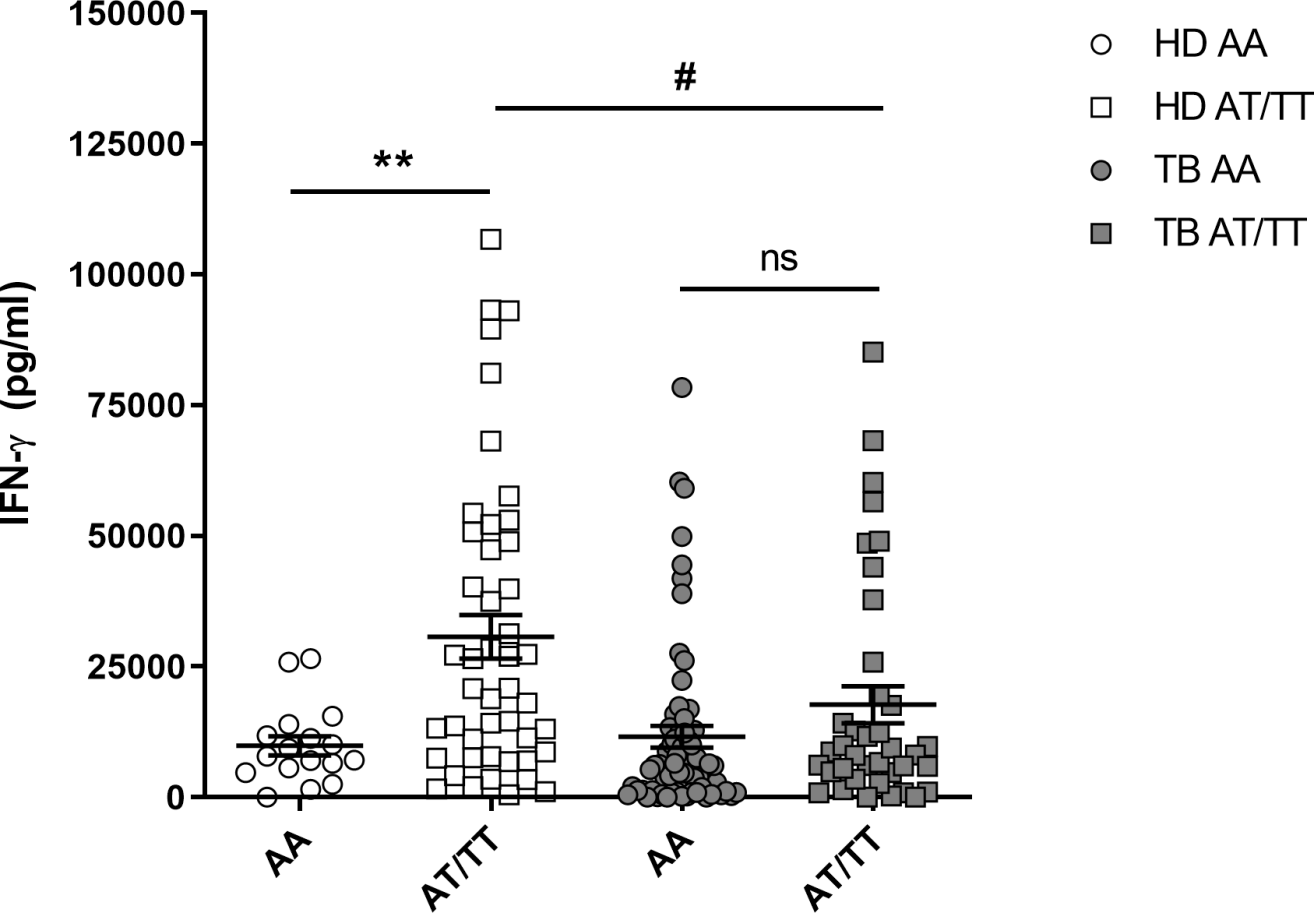
Production of IFN-γ by PBMCs from HD and TB patients carrying the genotypic variants of the IFN-γ gene (+874 A/T). PBMCs from healthy donors (HD, n = 63) and TB patients (TB, n = 102) carrying the different genotypes of the IFN-γ gene (+874 A/T) SNP were stimulated for five days with *M. tuberculosis*-Ag, and IFN-γ production was determined by ELISA. *P* values were calculated by one way ANOVA and Tukey’s multiple comparison test. ** p < 0.01 between HD genotypes. # p < 0.05 between HD and TB patients AT/TT genotypes. ns, not signficant.

We found that the presence of the T allele was associated with higher IFN-γ production (**Figure 2**), with significant differences only in HD. We also observed that HD with the AT and TT genotypes produced higher levels of IFN-γ than TB patients with the same genotypes (**Figure 2**). Finally, we did not find any association between the genotypes and the clinical data, which indicates that the +874 A/T IFNG SNP is not associated with TB severity in our study population (**Table 4**). The increased production of IFN-γ observed in HD suggests that other genetic and epigenetic factors could be involved in the regulation of IFN-γ against *M. tuberculosis*.

**Table 4:** Association between clinical parameters and the rs2430561 SNP genotypic variants during active tuberculosis.

TB patients	rs2430561 genotype			*P* value
	AA	AT	TT	
Hematologic Studies (n=91)				
**Leukocytes (cells/ml)**	10090 ( ± 491)	10436 ( ± 582)	9140 ( ± 1232)	>0,05^a^
**Lymphocytes (cells/ml)**	1525 ( ± 89)	1528 ( ± 102)	2112 ( ± 332)	>0,05^a^
**Monocytes (cells/ml)**	914 ( ± 48)	857 ( ± 53)	881 ( ± 156)	>0,05^a^
AFB in sputum smear (n=120)				
**BAAR- or BAAR+**	62 (84%)	28 (76%)	6 (67%)	0,3509^b^
**BAAR++ or BAAR+++**	12 (16%)	9 (24%)	3 (33%)	
Radiological Lesions (n=99)				
**Mild or Moderate**	26 (41%)	12 (41%)	2 (29%)	0,8033^b^
**Severe**	37 (59%)	17 (59%)	5 (71%)	
**Days of disease evolution (n=75)**	106,43 ( ± 13,22)	84,13 ( ± 10,62)	102,33 ( ± 22,18)	>0,05^a^

^a^ *P* values were calculated by the Tukey’s multiple comparisons test. ^b^ *P* values were calculated by the Chi-Square (χ2) test for categorical variables.

### Methylation status at the -53 CpG site of the promoter region of the IFN-γ gene

Methylation of the IFNG promoter could be a negative regulation mechanism during the immune response against *M. tuberculosis*. Therefore, methylation at the -53 CpG site of IFNG was evaluated by pyrosequencing of bisulfite-treated DNA from patients with active TB and HD. Individuals with latent tuberculosis (LTBI) were also included in this analysis. Although there is growing evidence of epigenetic modifications in TB (Kathirvel and Mahadevan, 2016), it is not yet fully understood how epigenetic alterations might trigger the activation from LTBI into active TB.

**Figure 3:**
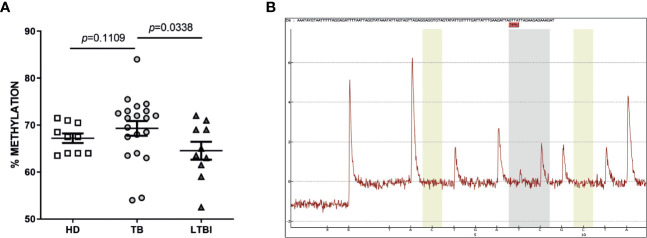
Methylation status of the -53 CpG of IFNG promoter in whole blood. Genomic DNA was obtained from whole blood from HD, TB patients and LTBI. Bisulfite converted-DNA was amplified by PCR and pyrosequencing was performed to determine the degree of methylation at the -53 CpG site of the IFNG promoter. **(A)** *P* values were calculated by one way ANOVA for non-parametric data and Dunn’s multiple comparison test. **(B)** A representative pyrogram is shown.

Interestingly, we observed a higher percentage of methylation of the CpG -53 site of the IFN-γ gene promoter region in TB patients compared to LTBI (TB = 69.30% vs. LTBI = 64.55%) (**Figures 3A, B**).

**Figure 4:**
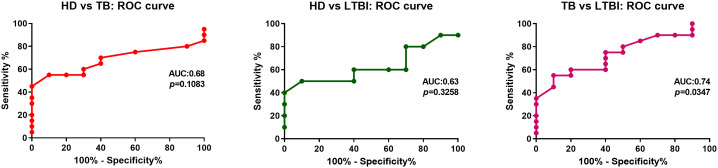
ROC curve analyses for evaluation of the predictive value of -53 CpG methylation on disease status. ROC curve analyses for evaluation of the predictive value of whole blood methylation levels at IFNG -53 CpG for differentiating HD individuals from TB, HD individual from LTBI and TB from LTBI. ROC, receiver operating characteristic; AUC, area under the ROC curve.

Moreover, we performed a ROC analysis for the methylation of IFNG, obtaining significant results for AUC analysis among TB vs LTBI individuals (**Figure 4**). Given that this kind of analysis plays a central role in evaluating diagnostic ability of tests to discriminate the true state of subjects (Hajian-Tilaki, 2013), our results could indicate a role for methylation at the -53 CpG site in disease reactivation.

## Discussion

Tuberculosis, an ancient disease, is still one of the biggest killers worldwide, with a high morbidity rate. It is known that only about 10% of the *M. tuberculosis*-infected population progresses to active tuberculosis which leads to the following question: Do patients with active TB have a genetic predisposition that makes them more susceptible to disease progression? Single nucleotide polymorphisms in several candidate genes, especially polymorphisms in cytokine genes are known to modulate cytokine levels, which may influence susceptibility to tuberculosis infection and disease. Given the essential role of IFN-γ to control mycobacterial replication and development of cellular immune response (Flesch and Kaufmann, 1987; Serbina et al., 2001; Salgame, 2005), our case-control study focused on determining whether rs2430561 SNP (+874 A/T) is associated with susceptibility to tuberculosis in Argentinean population.

In this study, we found a significant increase in the frequency of A allele (79%) and AA genotype (64%) in TB patients as compared with HD suggesting an association of the rs2430561 SNP A variant with susceptibility to tuberculosis disease in Argentina. Remarkably, more than 45% of individuals from the HD population (91 out of 199, QFT-GIT negative) were subjects exposed to *M. tuberculosis* (TB contacts or healthcare staff). Among them, 60.44% has a T allele, reinforcing that the presence of the T allele is indeed associated with resistance to *M. tuberculosis* infection. Despite Etokebe et al. did not find correlation between the +874 A/T polymorphism and tuberculosis disease in a Croatian population (Etokebe et al., 2006), our results are consistent with previous studies that have demonstrated an association of A allele and AA genotype with susceptibility to tuberculosis in Sicilian, Spanish, Hong Kong, Brazilians, South African and Egyptian populations (Lio et al., 2002; Lopez-Maderuelo et al., 2003; Rossouw et al., 2003; Tso et al., 2005; Amim et al., 2008; Mosaad et al., 2010).

A few reports have analyzed the association of rs2430561 SNP with the clinical manifestations of the disease (Lopez-Maderuelo et al., 2003; Etokebe et al., 2006; Ansari et al., 2011). Lopez-Maderuelo et al. showed an association of AA genotype with a far advanced form of radiographic extent of disease (Lopez-Maderuelo et al., 2003). Likewise, a study in a Pakistan population reported an overrepresentation of the T allele in less severe forms of pulmonary TB (Ansari et al., 2011). However, our results showed no association with clinical parameters demonstrating that IFN-γ +874 A/T polymorphism is associated with TB susceptibility but not with disease severity in our population of study.

We have previously demonstrated that low levels of IFN-γ are associated with disease severity (Pasquinelli et al., 2004; Jurado et al., 2012). Since it has been reported that +874 T to A polymorphism overlaps with a putative NF-κ B binding site which might have functional consequences for the transcription of the human IFN-γ gene (Pravica et al., 2000); we investigated the functional association of the different +874 genotypes with IFN-γ production. Our study demonstrates that *M. tuberculosis*-stimulated PBMCs from HD carrying the AA genotype produced strikingly significantly lower levels of IFN-γ than those carrying the AT and TT genotype. However, while a tendency was observed in TB patients, we did not find significant differences on the production of IFN-γ. On the other side, we did observe a significant difference in the production of IFN-γ between HD and TB patients carrying a T allele, which suggests that besides the presence of the T allele other mechanisms are regulating IFN-γ during active disease.

Indeed, genetic polymorphisms are not the only inheritable character of DNA that could influence susceptibility to disease. Epigenetic mechanisms such as DNA methylation and histone acetylation regulate the rate of transcription and/or specific tissue expression of certain genes without altering the DNA sequence.

Recent studies suggest that *M. tuberculosis* can alter the host epigenome to modulate the transcriptional machinery and plays a major role in modulating the immune response of the host (Kathirvel and Mahadevan, 2016). However, the mechanisms involved in epigenetic alterations during *M. tuberculosis* infection have not been fully understood yet. In addition to a potential causal role in pathogenic processes, epigenetic alterations – such as *de novo* methylation of DNA – can also occur as a direct or indirect consequence of disease and might serve as biomarkers of disease activity (Esterhuyse et al., 2012).

Owing to the major role of the epigenome in the phenotypic plasticity of the immune system and its ability to link environment and cellular phenotypes, host susceptibility to TB is expected to have an epigenetic predisposition component. In addition, epigenetic analyses will probably reveal changes at the epigenome resulting either directly or indirectly from infection. Growing evidence highlights the impact of pathogens genetic variation on susceptibility and severity of infections.

In Argentina, Monteserin et al. studied the genetic diversity of *M. tuberculosis* strains circulating in the Metropolitan Area of Buenos Aires (Monteserin et al., 2018). They showed that as in the rest of South America, genotypes of the Lineage 4 Euro-American (one of the seven globally described *M. tuberculosis* strains) predominate in our country and in contrast with the neighboring country Brazil, the RD^rio^ type (associated with high rate of transmission and drug resistance) does not play a major role in the TB epidemic (Monteserin et al., 2018). Moreover, the multidrug resistant M strain which belongs to the Lineage 4 Euro-American is highly prevalent in Argentina and has been shown to induce poor immune responses (Yokobori et al., 2022). However, the *M. tuberculosis* population structure in Argentina is still not fully comprehend.

It has been demonstrated that the IFNG promoter undergoes differential methylation during *in vitro* polarization of human peripheral blood T cells (Yano et al., 2003). This promoter is hypermethylated at the -53 CpG site during Th2 differentiation whereas it becomes hypomethylated in Th1 differentiated cells, existing a correlation between the degree of promoter methylation and the production of IFN-γ (Yano et al., 2003). Janson et al. also described different methylation levels at the -53 CpG site between human naive CD4 lymphocytes which were hypermethylated and Th1 lymphocytes which showed demethylation during differentiation (Janson et al., 2008). In other work, Dong et al. demonstrated that in human T cells early Th1-cell differentiation is accompanied by dynamic demethylation of CpGs at both the promoter and the CNS-1 regions of the IFNG locus (Dong et al., 2013). Moreover, epigenetic modifications in this locus were indispensable for the establishment of stable functional Th1 cytokine memory. The authors showed considerable levels of demethylation among different CpG of the promoter and the CNS1 region, including the -53 CpG site studied in our work (Dong et al., 2013).

In mice, naive T cells show a hypomethylated state of the IFNG promoter, which is maintained as they differentiate into Th1 cells (Winders et al., 2004). On the contrary, under Th2-polarizing conditions, CD4 cells display higher levels of methylation in the IFNG promoter, including the -53 CpG site (Winders et al., 2004). Besides, it has been shown that the methylation of the -53 CpG site impairs IFNG promoter activity and ATF2/c-Jun and CREB binding *in vitro* (Jones and Chen, 2006).

Despite the presence of some differences in the regulation of IFNG transcription between mice and human T lymphocytes, these data demonstrate the epigenetic involvement in immune regulation and evidence the -53 CpG site as an evolutionarily conserved position essential for IFN-γ production. When we evaluated the methylation status of the IFNG CpG -53 site in whole blood, our results showed increased methylation levels in patients with active TB compared to LTBI people. Surprisingly, we did not find differences in the methylation status between HD and TB patients, which could be attributed to a reactive epigenome. The lowest levels of methylation were observed for LTBI, who has the ability to control the infection. Considering that patients with active TB are not able to contain the infection at the time they were studied, a hypermethylation status could be expected.

Moreover, ROC and AUC analysis demonstrated that hypermethylation of this site could be a good biomarker of disease status; which reinforce the importance of this epigenetic modification as a key mechanism during active disease.

In accordance, some reports have shown changes in human methylome during TB or in response to BCG. Lyu et al. conclude that DNA methylation might be a promising TB diagnostic biomarker, as they identified TB-related targets differentially methylated by logistic regression and elastic net regression that were validated by bisulfite conversion and PCR (Lyu et al., 2022). DNA methylation has been also associated to anti-mycobacterial activity in PBMCs from BCG-vaccinated individuals (Verma et al., 2017). Recently, DiNardo et al. characterized DNA methylation of different genes that are critical to anti-mycobacterial immunity (DiNardo et al., 2020). They showed that immune cells from patients with active TB present a hypermethylated state, leading to nonspecific immune responsiveness (DiNardo et al., 2020).

These studies demonstrate the importance of evaluating the epigenome in the context of TB, however, none of them have evaluated the relevance of the -53 site in particular. Although the number of samples evaluated in the present study limits the extrapolation of our findings, since DNA methylome patterns are sensitive to environmental and seasonal factors, age, and ethnicity, it is attractive to focus on a well-defined study group as we did in our work. Another issue to highlight from our study is that we analyzed changes in the methylation of the -53 site in peripheral blood. Peripheral blood biomarkers, whether at the genomic, transcriptomic, epigenetic, or proteomic level, have received special attention in recent years. It is noteworthy that epigenetics can establish bridges between the host, *M. tuberculosis* and the environment. Therefore, epigenetic biomarkers in peripheral blood have great potential in the diagnosis and monitoring of progression to active TB. Epigenetic markers may provide a novel biosignature for discrimination between LTBI and active TB disease, for monitoring drug treatment outcome, and, in the long run, for predicting the risk of progression of LTBI to active TB disease (Esterhuyse et al., 2012).

To our knowledge, there are only three reports in South America population that study the association of the +874 A/T SNP with resistance/susceptibility to TB (Henao et al., 2006; Amim et al., 2008; Araujo et al., 2017). Recently, another IFN-γ SNP, the IFNG rs1861494, was associated with TB susceptibility in Argentinean population (Rolandelli et al., 2018). Our work, is the first to study association and functional relevance of rs1861494 SNP in Argentina. Even though we did not find correlation with the severity of the disease, we demonstrated that AA genotype and A allele are correlated with low IFN-γ production and increased susceptibility to develop active TB. Moreover, we also confirmed the methylation of -53 CpG of IFNG in TB patients. Many other polymorphic genes should be investigated in active and latent TB, but our results indicate that the AA genotype of the rs2430561 SNP could be a potential genetic biomarker for tuberculosis susceptibility in Argentina.

## Methods

### Healthy donors and patients

HIV-seronegative patients with active tuberculosis (TB) were evaluated at Dr. F. J. Muñiz Hospital (Buenos Aires, Argentina). Diagnosis of disease was established based on clinical and radiological data, identification of acid-fast bacilli in sputum, and isolation of *M. tuberculosis* in culture. Patients included in this study had received less than one week of anti-tuberculosis therapy. BCG-vaccinated healthy adults lacking a history of TB (household contacts and healthcare workers) were recruited at Argentinean Referral Hospitals. Among this group of individuals, diagnosis of LTBI was established using QuantiFERON-TB Gold In-Tube (QFT-GIT; Qiagen, USA; according to the manufacturer’s directions). LTBI diagnosis was assigned to any subject with a positive QFT-GIT and no clinical or radiological evidence of active TB. The group of healthy donors (HD) was comprised by adult individuals who had received BCG vaccination at birth and lacked a history of TB (tested by chest X-rays and analysis of acid-fast bacilli in sputum) and with negative QFT-GIT. Peripheral blood was collected in heparinized tubes from all individuals participating in the study after receiving informed consent. All methods were carried out in accordance with relevant guidelines and regulations. All experimental protocols were approved by a licensing committee from Hospital Dr. F. J. Muñiz (Buenos Aires, Argentina).

### *Mycobacterium tuberculosis* antigen

*In vitro* stimulation of cells throughout the study was performed with a cell lysate from the virulent *M. tuberculosis* H37Rv strain prepared by probe sonication (*Mtb*-Ag). The strain was obtained through BEI Resources, NIAID, NIH: *Mycobacterium tuberculosis*, Strain H37Rv, Whole cell lysate, NR-14822 (Bethesda, MD, USA).

### Cell preparation and reagents

Peripheral blood mononuclear cells (PBMCs) were isolated by centrifugation over Ficoll-Hypaque (GE Healthcare) and cultured (1 X 10^6^ cells/mL), with or without *Mtb*-Ag (10 µg/mL) with RPMI 1640 medium (Gibco) supplemented with L-glutamine (Sigma Aldrich), 10% Fetal Bovine Serum (Gibco), 100 U/mL of Penicillin and 100 µg/mL of Streptomycin (Gibco). After 5d, IFN-γ production was determined by ELISA (BioLegend).

### DNA extraction and genotyping

Genomic DNA was extracted from whole blood samples using the Quick-gDNA™ Blood MiniPrep (Zymo Reasearch) and buccal swabs (Biodynamics) of TB patients, and HD individuals according to the manufacturer’s instructions. Amplification refractory mutation system-polymerase chain reaction (ARMS-PCR) was performed for the rs2430561 SNP genotyping, as previously described (Pravica et al., 2000). The conditions included initial denaturation (95°C for 5min), 10 rounds for internal control amplification of 95°C for 30s, 62°C for 50s and 72°C for 60s; then 20 rounds of denaturation at 95°C for 20s, 56°C for 50s and 72°C for 50s with a final extension of 5min at 72°C. The amplified products were monitored by electrophoresis on a 2% agarose gel containing Syber Safe (Thermo).

### Methylation analysis of -53 CpG site in the IFNG promoter

Methylation analysis was carried out by bisulfite conversion of genomic DNA from whole blood samples of TB patients, HD and LTBI individuals. After extraction of genomic DNA from whole blood, bisulfite conversion was performed using the EZ DNA Methylation-Lightning™ Kit (Zymo Research) according to the manufacturer’s instructions. The IFNG promoter region was amplified by Bisulfite PCR using Go Taq Hot Start Polymerase (Promega) and the following primers designed with Methyl Primer Express: Fw 5´-TAAGGAGTTTAA AGGAAATTTTAATTATA-3´, Rv (biotinylated) 5´-ATCTTTCTCTTCTAATAACTAATCTTCAA- 3´). Following an initial denaturation of 94°C for 3min samples were subjected to 40 rounds of PCR consisting of 94°C for 30s, 48°C for 30s and 72°C for 60s with a final extension time of 10 min at 72°C. Methylation levels were determined by pyrosequencing (Service from Instituto de Genética Veterinaria (IGEVET) “Ing. Fernando Noel Dulout”, UNLP-CONICET) using an internal primer Fw: 5´-TTAAAAAATTTGTGA-3´. Pyrosequencing assays were performed according to the manufacturer’s instructions using the PSQ 96MA (Qiagen). The methylation levels were determined using the software PYROMARK Q96MA (Qiagen).

### Statistical analysis

The genotype and allele frequencies were obtained by direct counting. Hardy–Weinberg equilibrium was tested between cases/controls separately (χ2 goodness-of-fit test). Comparisons of the distributions of the allele and genotype frequencies between cases/controls were performed using the χ2 test for homogeneity. The association between the rs2430561 genotypes and the case/control condition was estimated as an odds ratio (OR). The quantitative data were expressed as mean ± standard error of the mean (SEM), and the Mann–Whitney U test or the Kruskal-Whallis (ANOVA) test for unpaired and non-parametric samples was used to analyze differences between groups. For categorical variables, the χ2 test was performed to compare proportions of subjects between groups. All statistical analysis were performed using GraphPad Prism v8.0 (GraphPad Software). p < 0.05 was considered statistically significant.

## Data availability statement

The original contributions presented in the study are included in the article/**Supplementary Materials**, further inquiries can be directed to the corresponding author/s.

## Ethics statement

The studies involving human participants were reviewed and approved by Hospital Dr. F. J. Muñiz. The patients/participants provided their written informed consent to participate in this study.

## Author contributions

GA, RH, AB, VG and VP contributed to the conception and design of the study. GA and RH performed the experiments and analyzed the data. AB, ME and JC contributed with some of the experiments and provided methodological support and insightful data discussion. RH, AB and VP wrote the first draft of the manuscript. All authors contributed to manuscript revision, read, and approved the submitted version. DP and RM were in charge of patients’ diagnosis, obtained the blood samples and contributed with the analysis of the clinical data. All authors contributed to the article and approved the submitted version.
