# Supplementary material for: Association of IFN-γ +874 A/T SNP and hypermethylation of the -53 CpG site with tuberculosis susceptibility
Source: Front Cell Infect Microbiol. 2023 Jan 19;13:1080100. doi: 10.3389/fcimb.2023.1080100 (PMC9892940; doi:10.3389/fcimb.2023.1080100)
Supplement: Supplementary file 1 [file Table_1.docx]

# Supplementary Tables.

**Table SI.** Analyses of each genotype stratified by sex.

| **Genotype** | **HD = 199** | | | ***P* Value** |
| --- | --- | --- | --- | --- |
|  | Male = 68 | Female = 126 | No data = 5 | Male vs Female |
| **AA** | 20 (29.41) | 44 (34.92) | 3 (60.00) | 0.7381 |
| **AT** | 35 (51.47) | 60 (47.62) | 1 (20.00) |  |
| **TT** | 13 (19.12) | 22 (17.46) | 1 (20.00) |  |
|  |  |  |  |  |
| **Genotype** | **TB = 173** | | | ***P* Value** |
|  | Male = 133 | Female = 26 | No data = 14 | Male vs Female |
| **AA** | 87 (65.42) | 15 (57.69) | 8 (57.14) | 0.1768 |
| **AT** | 39 (29.32) | 7 (26.92) | 6 (42.86) |  |
| **TT** | 7 (5.26) | 4 (15.39) | 0 (0.00) |  |
